# Chronic pain and COVID-19 hospitalisation and mortality: a UK Biobank cohort study

**DOI:** 10.1097/j.pain.0000000000002663

**Published:** 2022-04-22

**Authors:** Claire E. Hastie, Hamish M.E. Foster, Bhautesh D. Jani, Catherine A. O'Donnell, Frederick K. Ho, Jill P. Pell, Naveed Sattar, Srinivasa V. Katikireddi, Frances S. Mair, Barbara I. Nicholl

**Affiliations:** a Public Health, Institute of Health and Wellbeing, University of Glasgow, Glasgow, United Kingdom; b General Practice and Primary Care, Institute of Health and Wellbeing, University of Glasgow, Glasgow, United Kingdom; c British Heart Foundation Glasgow Cardiovascular Research Centre, Institute of Cardiovascular and Medical Sciences, University of Glasgow, Glasgow, United Kingdom; d MRC/CSO Social and Public Health Sciences Unit, Institute of Health and Wellbeing, University of Glasgow, Glasgow, United Kingdom

**Keywords:** Chronic pain, COVID-19, hospitalisation, mortality

## Abstract

The risk of COVID-19 in those with chronic pain is unknown. We investigated whether self-reported chronic pain was associated with COVID-19 hospitalisation or mortality. UK Biobank recruited 502,624 participants aged 37 to 73 years between 2006 and 2010. Baseline exposure data, including chronic pain (>3 months, in at least 1 of 7 prespecified body sites) and chronic widespread pain (>3 months, all over body), were linked to COVID-19 hospitalisations or mortality. Univariable or multivariable Poisson regression analyses were performed on the association between chronic pain and COVID-19 hospitalisation and Cox regression analyses of the associations with COVID-19 mortality. Multivariable analyses adjusted incrementally for sociodemographic confounders, then lifestyle risk factors, and finally long-term condition count. Of 441,403 UK Biobank participants with complete data, 3180 (0.7%) were hospitalised for COVID-19 and 1040 (0.2%) died from COVID-19. Chronic pain was associated with hospital admission for COVID-19 even after adjustment for all covariates (incidence rate ratio 1.16; 95% confidence interval [CI] 1.08-1.24; *P* < 0.001), as was chronic widespread pain (incidence rate ratio 1.33; 95% CI 1.06-1.66; *P* = 0.012). There was clear evidence of a dose-response relationship with number of pain sites (fully adjusted global *P*-value < 0.001). After adjustment for all covariates, there was no association between chronic pain (HR 1.01; 95% CI 0.89-1.15; *P* = 0.834) but attenuated association with chronic widespread pain (HR 1.50, 95% CI 1.04-2.16, *P*-value = 0.032) and COVID-19 mortality. Chronic pain is associated with higher risk of hospitalisation for COVID-19, but the association with mortality is unclear. Future research is required to investigate these findings further and determine whether pain is associated with long COVID.

## Abstract 2

Supplemental Digital Content is Available in the Text.

Chronic pain is associated with increased risk of hospitalisation due to COVID-19, and this risk increases with the number of pain sites.

## 1. Introduction

Chronic pain significantly affects well-being, with chronic low back pain being the leading cause of disability globally.^15^ In the United Kingdom, the prevalence of chronic pain in adults is estimated to be between 35.0% and 51.3%.^11^ Globally, chronic pain affects 1 in 5 adults, although this is believed to be an underestimate.^22^ Chronic pain is a complex condition which is challenging to treat.^32,35^ The burden of disease due to chronic pain and demand for effective pain management is likely to increase during the COVID-19 pandemic, with joint and muscle pain being reported both in the acute phase and by those with long COVID.^3,30^ Furthermore, pre-existing musculoskeletal pain can be exacerbated by COVID-19.^12^ Several authors have commented on the implications of COVID-19 for pain management^7,42,44^ including the use of telemedicine to minimise face-to-face encounters and reduce SARS-CoV-2 transmission.^9,16,29,47^ Although it is clear that chronic pain can be a result of SARS-CoV-2 infection, it remains unclear whether chronic pain may predispose to more severe SARS-CoV-2 infection or higher risk of adverse outcome.

To mitigate the impact of COVID-19 and target public health and clinical interventions, many hospital and community studies have tried to identify risk factors for COVID-19 outcomes. Sociodemographic characteristics such as older age, socioeconomic deprivation, male sex, and Black and South Asian ethnicity have consistently been associated with higher risk of SARS-CoV-2 infection and death in the United Kingdom.^21,34,40,51^ The association between ethnicity and COVID-19 has been shown to be modified by occupation, although other factors including overcrowded housing, poorer socioeconomic status, and structural racism are also important.^18,26,33,37^ Some individual long-term conditions (LTCs), for example, cardiac disease, diabetes, and obesity, have been associated with higher COVID-19 case fatality.^8,34^ Furthermore, multimorbidity (the presence of 2 or more LTCs) is also associated with increased risk of SARS-CoV-2 infection^34^ as is frailty.^41^

Chronic pain is more likely to be reported by those of Asian and Black ethnicity^39^ and in those of low socioeconomic status.^20^ A study of multimorbidity in primary care found that pain was associated with all the 10 most prevalent multimorbidity clusters.^4^ Research into whether chronic pain is associated with SARS-CoV-2 infection or COVID-19 mortality is limited. A recent study observed that, in children and adults with sickle cell disease, those with frequent prior acute care visits for pain were more likely to be hospitalised during their COVID-19 illness than those without such a history.^36^ However, it is not known whether this relationship exists in the general population.

We explored whether UK Biobank participants with self-reported chronic pain had a higher risk of COVID-19 hospitalisation and mortality.

## 2. Methods

### 2.1. Participants

UK Biobank is a prospective cohort study. It recruited 502,624 participants aged 37 to 73 years across England, Scotland, and Wales between 2006 and 2010. At baseline, touch-screen questionnaires and nurse-administered interviews were used to collect demographic, health, environmental, and lifestyle data using standardised protocols and to record biological measurements.^6,45^ This study received ethical approval from the North West Multi-Centre Research Ethics Committee (REC reference: 16/NW/0274). All participants gave written informed consent for data collection, analysis, and record linkage.

### 2.2. Definitions

Baseline data from UK Biobank were linked, both prospectively and retrospectively, to hospital inpatient data, comprising Hospital Episode Statistics (HES) Admitted Patient Care data for England and Scottish Morbidity Record (SMR01) data for Scotland. Potential participants were excluded if their baseline assessment centre was in Wales because Welsh hospital inpatient data have not been updated to include the pandemic period.

Primary outcome was COVID-19 hospitalisation, defined as *ICD* code U071 (virus identified). Secondary outcome was COVID-19 mortality, defined as *ICD* code U071 or U072 (virus probable, suspected, or clinically epidemiologically diagnosed) recorded as the primary cause of death.

The hospital data included admission date and COVID-19 diagnosis. For those who had a COVID-19 diagnosis, admissions data were available between 15 of August 2019 and 26 of March 2021. Data were linked to Death Register Data for England and Scotland (available from 5 March 2020 to 17 March 2021). All COVID-19 deaths were included regardless of where they occurred: in the community and during or after admission to hospital.

Chronic pain, self-reported at baseline (2006-2010), using a touch-screen questionnaire, was defined as having pain for at least 3 months in one or more of the following sites: head, face, neck/shoulder, back, abdomen, hip, and knee. A binary yes/no variable was constructed for chronic pain. The total number of sites of chronic pain was categorised as none (no chronic pain), 1, 2 to 3, or 4 to 7. Chronic widespread pain was defined as self-reported pain all over the body for at least 3 months. It was analysed as a separate exposure, comparing participants who reported chronic widespread pain with those without chronic pain.

Ethnicity was self-reported and categorised as White, Black, South Asian, Chinese, mixed, or other. Current age was calculated as age at assessment plus the number of years between assessment date and date of death or censor date. Current age was entered into all analyses as a continuous variable. Body mass index (BMI) was derived from weight (kg)/height (m)^2^ and categorised into underweight (<18.5 kg/m^2^), normal weight (18.5-24.9 kg/m^2^), overweight (≥25-29.9 kg/m^2^), obese (≥30-34.9 kg/m^2^), and morbidly obese (≥35 kg/m^2^).^50^ Area-level socioeconomic deprivation was assessed using the Townsend deprivation index, which incorporates unemployment, car ownership, home ownership, and household overcrowding.^48^ Higher Townsend scores represent greater socioeconomic deprivation; scores were categorised into quintiles within the study sample. Smoking status, frequency of alcohol consumption, and physical activity were self-reported. Smoking status was categorised as never or current or previous smoker. Alcohol consumption was categorised into never or special occasions only, 1 to 3 times a month, 1 to 4 times a week, and daily or almost daily. Physical activity was categorised into none, low, medium, or high using Metabolic Equivalent Task (MET) scores based on the International Physical Activity Questionnaire (IPAQ short form) scoring protocol.^23^

Number of LTCs was defined as a count of 43 self-reported conditions which have previously been used in UK Biobank studies of multimorbidity (Supplementary Table 1, available at http://links.lww.com/PAIN/B629).^24^ This list of conditions is based on previously published literature on multimorbidity.^1,38^ For chronic pain as a binary and ordinal variable, the “painful conditions” grouping contained only trigeminal neuralgia, shingles, and headache. For chronic widespread pain, the “painful conditions” grouping also contained back pain/problems, sciatica/disc/nerve problems, plantar fasciitis, carpal tunnel syndrome, and joint osteo/spine arthritis/spondylitis/arthritis with no other symptoms. LTC count was entered into models categorised as 0, 1, 2 to 3, and ≥4 LTCs.

### 2.3. Statistical analyses

Univariable Poisson regression analysis was performed for the association between chronic pain and COVID-19 diagnosis (in hospital). In multivariable analysis, the model was first adjusted for sociodemographic factors (current age, sex, Townsend deprivation quintile, ethnicity, and assessment centre location), then additionally adjusted for lifestyle factors (smoking status, alcohol frequency, BMI, and physical activity), and finally for number of LTCs. The adjustment for number of LTCs was performed last because LTCs can be considered as a confounder or a mediator^10,49^ (or both) in the relationship between chronic pain and COVID-19 diagnosis and mortality. Chronic pain was coded first as a binary variable, then as an ordinal variable derived from number of pain sites (none, 1, 2-3, and 4-7), and finally as chronic widespread pain vs no chronic pain.

Cox proportional hazards regression was performed for the association between chronic pain and COVID-19 mortality (deaths in the whole study sample), and for the association between chronic pain and COVID-19 case fatality (deaths among those hospitalised for COVID-19), both adjusted as above. Models included time to event (mortality or end of follow-up) from baseline assessment. The proportional hazards assumption was assessed through formal tests of Schoenfeld residuals. Analyses were undertaken using Stata v14.

## 3. Results

From an initial sample of 502,503 UK Biobank participants, 28,806 were excluded because they died before the first hospital admission for COVID-19, and a further 19,438 were excluded because they attended a Welsh assessment centre (Supplementary Fig. 1, available at http://links.lww.com/PAIN/B629). Of the remainder, complete data on chronic pain and covariates were available for 441,403 participants. Of these, 3180 (0.7%) were admitted with a COVID-19 diagnosis and there were 1040 (0.2%) COVID-19–related deaths. 1724 (54%) of those hospitalised for COVID-19 had a history of chronic pain, as did 539 (52%) of those whose death was related to COVID-19.

Table 1 presents participant characteristics by COVID-19 hospitalisation. Among participants with a positive COVID-19 test in hospital, 54% had self-reported chronic pain in at least 1 site at baseline, compared with 43% of those without a positive test. Presence of chronic pain was associated with SARS-CoV-2 infection univariably (Table 2; IRR 1.57, 95% CI 1.46-1.68, *P* < 0.001); the association was attenuated after adjustment for potential confounders (adjusted IRR 1.25, 95% CI 1.17-1.35; *P* < 0.001) and further attenuated but remained significant after adjustment for LTCs (fully adjusted IRR 1.16, 95% CI 1.08-1.24; *P* < 0.001).

**Table 1:** Characteristics of study population by hospitalisation for COVID-19.

	Hospitalisation for COVID-19		
	No	Yes	
	N (%)	N (%)	χ^2^ test *P*
Chronic pain			
No	249,090 (57.03)	1456 (45.79)	<0.001
Yes	188,314 (42.97)	1724 (54.21)	
Sex			
Female	242,914 (55.43)	1315 (41.35)	<0.001
Male	195,309 (44.57)	1865 (58.65)	
Self-reported ethnicity			
White	414,543 (94.60)	2845 (89.47)	<0.001
Black	7185 (1.64)	138 (4.34)	
South Asian	8504 (1.94)	123 (3.87)	
Chinese	1406 (0.32)	16 (0.50)	
Mixed	2621 (0.60)	15 (0.47)	
Other	3964 (0.90)	43 (1.35)	
Townsend deprivation quintile			
1 (least deprived)	89,252 (20.37)	431 (13.55)	<0.001
2	88,709 (20.24)	495 (15.57)	
3	87,787 (20.03)	522 (16.42)	
4	87,641 (20.00)	668 (21.01)	
5 (most deprived)	84,834 (19.36)	1064 (33.46)	
BMI category			
Underweight	2179 (0.50)	11 (0.35)	<0.001
Normal weight	141,324 (32.25)	515 (16.19)	
Overweight	188,632 (43.04)	1280 (40.25)	
Obese	76,777 (17.52)	855 (26.89)	
Morbidly obese	29,311 (6.69)	519 (16.32)	
Smoking status			
Never	244,872 (55.88)	1361 (42.80)	<0.001
Current/previous	193,351 (44.12)	1819 (57.20)	
Alcohol consumption frequency			
Never/special occasions	82,979 (18.94)	962 (30.25)	<0.001
1-3 times a month	49,270 (11.24)	351 (11.04)	
1-4 times a week	216,820 (49.48)	1328 (41.76)	
Daily or almost daily	89,154 (20.34)	539 (16.95)	
Physical activity			
High	46,218 (10.55)	180 (5.66)	<0.001
Medium	349,605 (79.78)	2411 (75.82)	
Low	15,868 (3.62)	189 (5.94)	
None	26,532 (6.05)	400 (12.58)	
Number of long-term conditions			
0	175,807 (40.12)	703 (22.11)	<0.001
1	146,912 (33.52)	956 (30.06)	
2-3	101,907 (23.25)	1230 (38.68)	
≥4	13,597 (3.10)	291 (9.15)	

	Median (IQR)	Median (IQR)	Wilcoxon *P*
Current age (years)	69 (62-75)	73 (66-78)	<0.001

BMI, body mass index; IQR, interquartile range; N, number.

**Table 2:** Poisson and Cox regression models of the associations between chronic pain status and COVID-19 hospitalisation, mortality, and case fatality.

	Chronic pain	Univariable	Multivariable 1*	Multivariable 2†	Multivariable 3‡
COVID-19 hospitalisation, IRR (95% CI)	No (N = 251,365)	1	1	1	1
	Yes (N = 190,038)	1.57 (1.46-1.68) *P* < 0.001	1.47 (1.37-1.58) *P <* 0.001	1.25 (1.17-1.35) *P <* 0.001	1.16 (1.08-1.24) *P <* 0.001
COVID-19 mortality, HR (95% CI)	No (N = 251,365)	1	1	1	1
	Yes (N = 190,038)	1.40 (1.24-1.59) *P <* 0.001	1.32 (1.16-1.49) *P <* 0.001	1.09 (0.96-1.23) *P* = 0.196	1.01 (0.89-1.15) *P* = 0.834
COVID-19 case fatality, HR (95% CI)	No (N = 1456)	1	1	1	1
	Yes (N = 1724)	0.94 (0.81-1.08) *P* = 0.349	0.97 (0.84-1.12) *P* = 0.669	0.92 (0.79-1.06) *P* = 0.234	0.91 (0.79-1.06) *P* = 0.221

* Adjusted for age, sex, Townsend score, ethnicity, and assessment centre location.

† Additionally adjusted for smoking status, alcohol intake frequency, BMI, and physical activity.

‡ Additionally adjusted for number of long-term conditions.

CI, confidence interval; HR, hazard ratio; IRR, incidence rate ratio.

When chronic pain was treated as an ordinal variable, there was a positive association between number of pain sites and COVID-19 with evidence of a dose-response relationship (Table 3): compared with no pain, 1 site IRR 1.21, 95% CI 1.10 to 1.32; 2 to 3 sites IRR 1.74, 95% CI 1.59 to 1.91; 4 to 7 sites IRR 2.83, 95% CI 2.45 to 3.28; global *P*-value<0.001). Adjustment for potential confounders and LTCs attenuated this association (compared with no pain, 1 site IRR 1.02, 95% CI 0.93-1.12; 2-3 sites IRR 1.23, 95% CI 1.12-1.35; 4-7 sites IRR 1.49, 95% CI 1.28-1.74; global *P*-value<0.001).

**Table 3:** Poisson and Cox regression models of the association between chronic pain category and COVID-19 hospitalisation, mortality, and case fatality.

	Number of chronic pain sites	Univariable	Multivariable 1*	Multivariable 2†	Multivariable 3‡
COVID-19 hospitalisation, IRR (95% CI)	0 (N = 251,365)	1	1	1	1
	1 (N = 101,609)	1.21 (1.10-1.32) *P <* 0.001	1.16 (1.06-1.27) *P =* 0.001	1.06 (0.97-1.16) *P* = 0.198	1.02 (0.93-1.12) *P* = 0.618
	2-3 (N = 70,422)	1.74 (1.59-1.91) *P* < 0.001	1.64 (1.49-1.79) *P* < 0.001	1.36 (1.24-1.49) *P* < 0.001	1.23 (1.12-1.35) *P* < 0.001
	4-7 (N = 12,495)	2.83 (2.45-3.28) *P* < 0.001	2.56 (2.20-2.96) *P* < 0.001	1.81 (1.55-2.10) *P* < 0.001	1.49 (1.28-1.74) *P* < 0.001
	Global test	*P* < 0.001	*P* < 0.001	*P* < 0.001	*P* < 0.001
COVID-19 mortality, HR (95% CI)	0 (N = 251,365)	1	1	1	1
	1 (N = 101,609)	1.15 (0.99-1.34) *P* = 0.077	1.09 (0.94-1.28) *P* = 0.260	0.980 (0.84-1.15) *P* = 0.798	0.95 (0.81-1.11) *P* = 0.513
	2-3 (N = 70,422)	1.45 (1.23-1.70) *P* < 0.001	1.36 (1.15-1.60) *P* < 0.001	1.08 (0.92-1.28) *P* = 0.336	0.999 (0.84-1.18) *P* = 0.989
	4-7 (N = 12,495)	2.40 (1.84-3.13) *P* < 0.001	2.30 (1.77-3.01) *P* < 0.001	1.51 (1.15-1.99) *P* = 0.003	1.30 (0.99-1.72) *P* = 0.062
	Global test	*P* < 0.001	*P* < 0.001	*P* = 0.019	*P* = 0.200
COVID-19 case fatality, HR (95% CI)	0 (N = 1456)	1	1	1	1
	1 (N = 710)	0.99 (0.83-1.18) *P* = 0.897	0.95 (0.79-1.14) *P* = 0.607	0.94 (0.78-1.12) *P* = 0.481	0.93 (0.78-1.12) *P* = 0.463
	2-3 (N = 711)	0.87 (0.72-1.04) *P* = 0.133	0.91 (0.75-1.09) *P* = 0.313	0.85 (0.70-1.03) *P* = 0.090	0.85 (0.70-1.03) *P* = 0.089
	4-7 (N = 205)	0.93 (0.70-1.24) *P* = 0.643	1.16 (0.87-1.56) *P* = 0.309	1.01 (0.75-1.37) *P* = 0.929	1.03 (0.76-1.39) *P* = 0.863
	Global test	*P* = 0.486	*P* = 0.429	*P* = 0.366	*P* = 0.341

* Adjusted for age, sex, Townsend score, ethnicity, and assessment centre location.

† Additionally adjusted for smoking status, alcohol intake frequency, BMI, and physical activity.

‡ Additionally adjusted for number of long-term conditions.

CI, confidence interval; HR, hazard ratio; IRR, incidence rate ratio.

Chronic widespread pain was associated with COVID-19 infection univariably (Table 4; IRR 3.07, 95% CI 2.50-3.77, *P* < 0.001); the association was attenuated after adjustment for potential confounders (adjusted IRR 1.68, 95% CI 1.36-2.08; *P* < 0.001) and further attenuated but remained significant after adjustment for LTCs (fully adjusted IRR 1.33, 95% CI 1.06-1.66; *P* = 0.012).

**Table 4:** Poisson and Cox regression models of the association between chronic widespread pain status and COVID-19 hospitalisation, mortality, and case fatality.

	Chronic widespread pain	Univariable	Multivariable 1*	Multivariable 2†	Multivariable 3‡
COVID-19 hospitalisation, IRR (95% CI)	No (N = 251,365)	1	1	1	1
	Yes (N = 5512)	3.07 (2.50-3.77) *P* < 0.001	2.51 (2.03-3.09) *P <* 0.001	1.68 (1.36-2.08) *P <* 0.001	1.33 (1.06-1.66) *P* = 0.012
COVID-19 mortality, HR (95% CI)	No (N = 251,365)	1	1	1	1
	Yes (N = 5512)	3.14 (2.24-4.41) *P <* 0.001	2.74 (1.94-3.87) *P <* 0.001	1.84 (1.29-2.62) *P* = 0.001	1.50 (1.04-2.16) *P* = 0.032
COVID-19 case fatality, HR (95% CI)	No (N = 1456)	1	1	1	1
	Yes (N = 98)	1.05 (0.72-1.51) *P* = 0.811	1.27 (0.87-1.86) *P* = 0.223	1.32 (0.90-1.94) *P* = 0.162	1.34 (0.90-1.99) *P* = 0.150

* Adjusted for age, sex, Townsend score, ethnicity, and assessment centre location.

† Additionally adjusted for smoking status, alcohol intake frequency, BMI, and physical activity.

‡ Additionally adjusted for number of long-term conditions.

CI, confidence interval; HR, hazard ratio; IRR, incidence rate ratio.

There was an association between pain and COVID-19 mortality univariably (Table 2; HR 1.40, 95% CI 1.24-1.59, *P*-value < 0.001), but this was fully attenuated after adjustment for confounders and LTCs (adjusted HR 1.01, 95% CI 0.89-1.15, *P*-value = 0.834). The pattern was similar when chronic pain was treated as an ordinal variable (Table 3; unadjusted compared with no pain, 1 site HR 1.15, 95% CI 0.99-1.34; 2-3 sites HR 1.45, 95% CI 1.23-1.70; 4-7 sites HR 2.40, 95% CI 1.84-3.13; global *P*-value < 0.001). The dose-response relationship remained and the global *P*-value was still significant after adjustment for confounders (adjusted, compared with no pain 1 site HR 0.980, 95% CI 0.84-1.15; 2-3 sites HR 1.08, 95% CI 0.92-1.28; 4-7 sites HR 1.51, 95% CI 1.15-1.99; global *P*-value = 0.019). This was no longer the case after adjustment for LTCs (global *P*-value = 0.200). Chronic widespread pain was associated with COVID-19 mortality univariably (Table 4; HR 3.14, 95% CI 2.24-4.41, *P*-value < 0.001). However, the association was attenuated after adjustment for confounders (HR 1.84, 95% CI 1.29-2.62, *P*-value = 0.001) and further attenuated after adjustment for LTCs (HR 1.50, 95% CI 1.04-2.16, *P*-value = 0.032). There was no evidence of violation of the proportional hazards assumption.

There was no clear association between chronic pain and COVID-19 case fatality. For the exposures, chronic pain status (Table 2) and number of pain sites (Table 3) hazard ratios were close to 1. For chronic widespread pain, there was a suggestion of increased case fatality but 95% confidence intervals were very wide (Table 4; fully adjusted HR 1.34, 95% CI 0.90-1.99, *P*-value=0.150). This may reflect the rarity of the outcome rather than there being no effect.

## 4. Discussion

To the best of our knowledge, this is the first study to explore the associations between chronic pain and COVID-19 hospitalisation and mortality in the general population. Chronic pain, both at individual sites and widespread, was associated with COVID-19 hospitalisation independent of potential confounders including presence of LTCs, and there was a clear dose-response relationship with the number of pain sites.

Any association between pain and COVID-19 mortality was less clear, suggesting that pain increases the risk of serious illness but may not increase the risk of what happens thereafter.

The effect sizes observed imply potentially small increases in the risk of COVID-19 hospitalisation at a population level. For example, the lower limit of the fully adjusted 95% confidence interval when chronic pain is treated as a binary variable represents only a relative increased risk of 8% when compared with those without chronic pain. However, those with pain at 4 to 7 sites are between 28% and 74% more likely to have COVID-19 infection compared with those with no pain, with a best estimate of 49%. This is important in clinical terms.

The relationship between chronic pain and hospitalisation for COVID-19 infection is currently unclear. One possibility is that chronic pain is a proxy measure for unmeasured risk factors or undiagnosed disease. It has been linked to a wide range of pathophysiological consequences, including effects on mood, cognition, sleep, cardiovascular risk, quality of life, and general functioning.^13^ Chronic widespread pain is associated with poorer self-reported general health,^46^ and people with chronic pain have higher rates of anxiety and depression.^19^ Alternatively, inflammatory mechanisms underpinning some chronic pain conditions^5^ may also make individuals susceptible to more severe COVID-19 infection, requiring hospitalisation, as may also be the case for other conditions where inflammation seems to be causally related, eg, cardiovascular disease.^27^ Furthermore, diminished physical functioning due to chronic pain may predispose to complications of COVID-19. For example, greater immobility due to chronic pain may potentiate thromboembolic complications and poorer preinfection cardiorespiratory function.

The strengths of UK Biobank include its large sample size and extensive phenotyping, which enabled us to adjust for a wide range of potential confounders including comorbidities and sociodemographic and lifestyle risk factors. Nonetheless, as with all observational studies, residual confounding because of unknown or unmeasured confounders is possible, for example, differential access to testing or hospitalisation. UK Biobank is not representative of the general population (response rate 5.5%)^14^ because its participants are less socioeconomically deprived, have fewer self-reported health conditions, and are less ethnically diverse.^14^ Nonetheless, estimates of effect size are expected to be generalisable and comparable with those obtained from more representative general population studies.^2^ Although we were able to measure the number of LTCs, we did not have data on their severity. Adjustment for LTC severity could have produced different estimates to our final models (adjusted for a simple LTC count) if those with severe LTCs have higher risk of adverse COVID-19 outcomes compared with those with only mild LTCs.

Data on chronic pain were collected before the COVID-19 pandemic excluding the possibility of reverse causation. In this analysis, we assumed that most of the UK Biobank participants who reported chronic pain at baseline continued to experience persistent pain over the medium to long term albeit that it may fluctuate regarding intensity and interference.^17^ Although pain status may, in reality, have changed over the intervening period, this is unlikely to have introduced systematic error. Studies that measure duration of chronic pain show that it persists, at least in the medium term, for 5 to 7 years.^28,31,43^ Kamaleri and colleagues showed that the number of pain sites (not specifically chronic pain) a person reported remained stable over a 14-year period^25^; a similar time frame from UK Biobank baseline assessments to the COVID-19 pandemic (10-14 years). Therefore, any change in pain status over time is likely to take the form of missing data on new onset pain which would likely result in an underestimate of the real effect.

The availability of testing and the testing strategies used have changed over the course of the pandemic. Therefore, incomplete ascertainment of infections is possible. Our ascertainment method should be relatively robust against changes in test strategy because we limited outcomes to COVID-19 hospitalisations and mortality. This excludes people tested in the community who were likely to have had milder symptoms, were more likely to have been denied access to testing, and experienced more dramatic temporal changes in access to testing.

## 5. Conclusion

Our analyses of UK Biobank data demonstrated an association between chronic pain (at separate sites or all over the body) and COVID-19 hospitalisation, independent of known LTCs, and clear evidence of a dose-response relationship with number of pain sites. Pain may be a proxy measure of undiagnosed underlying physical or mental health disease processes. Future studies are needed to corroborate our novel findings, investigate the underlying mechanisms, and investigate whether pre-existing pain is also an independent risk factor for long COVID.

## Conflict of interest statement

The authors have no conflicts of interest to declare.

## Appendix A. Supplemental digital content

Supplemental digital content associated with this article can be found online at http://links.lww.com/PAIN/B629.

## Supplemental video content

A video abstract associated with this article can be found at http://links.lww.com/PAIN/B630.
