# Supplementary material for: Chronic pain and COVID-19 hospitalisation and mortality: a UK Biobank cohort study
Source: Pain. 2022 Apr 22;164(1):84–90. doi: 10.1097/j.pain.0000000000002663 (PMC9756431; doi:10.1097/j.pain.0000000000002663)

Supplementary Table 1: 43 conditions comprising number of LTCs count

painful conditions in chronic pain analysis: trigeminal neuralgia/shingles/headache<br>additional painful conditions in widespread pain analysis: back pain/problems, sciatica/disc/nerve problems, plantar fasciitis, carpal tunnel syndrome, and joint osteo/spine arthritis/spondylitis/arthritis with no other symptoms
migraine
chronic fatigue syndrome
irritable bowel syndrome
rheumatoid arthritis/ankylosing spondylitis/other connective tissue disorder
chronic obstructive pulmonary disease
diverticular disease
dyspepsia
Parkinson’s disease
depression
endometriosis
peripheral vascular disease
alcohol problems
chronic sinusitis
chronic kidney disease
Meniere’s disease
osteoporosis
coronary heart disease
polycystic ovary syndrome
anxiety
stroke/transient ischemic attack
psychoactive substance misuse
pernicious anaemia
chronic liver disease
constipation
diabetes
multiple sclerosis
irritable bowel disorder
thyroid disorder
asthma
anorexia/bulimia
hypertension
epilepsy
schizophrenia
heart failure
glaucoma
prostate disease
atrial fibrillation
chronic bronchiectasis
viral hepatitis
cancer
psoriasis/eczema
dementia

Supplementary Figure 1: Participant flowchart

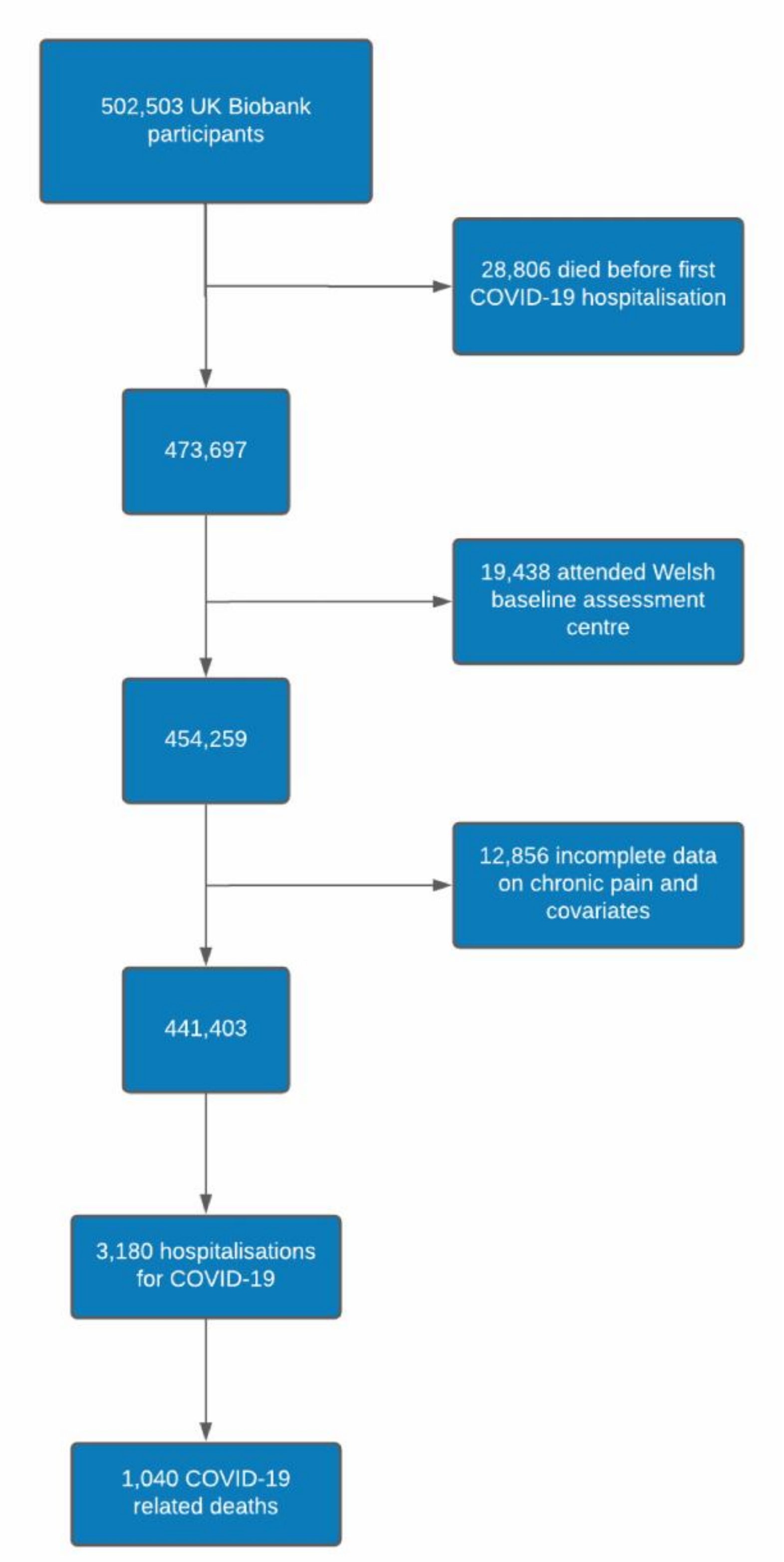

Supplement: Supplementary file 1 [file jop-164-084-s001.pdf]
